# Pulmonary Tuberculosis

**Published:** 1894-01-20

**Authors:** 


### PULMONARY TUBERCULOSIS (*continued*).

### Recent Remedies.

*Creosote.*—The employment of creosote and guaiacol have been so long in general use that they cannot be called new remedies, but new modifications in the method of administering these and other similar drugs are being continually introduced, and more especially methods by which the advantages presented by several bactericidal remedies are combined and obtained without involving the drawbacks inherent to the administration of the simple and crude drugs.

Creosote pills have been used by Dr. Albert Albu and Wehl[4] at the Moabit Hospital, Berlin, for more than five years. The dose has been increased until half a drachm and more has been given daily. The creosote is given in pills, each pill containing five-sixths of a grain of creosote. As many as fifty or even sixty of these pills have been given daily for weeks and months at a time. Yet among all his numerous cases Dr. Albu had none in which the number of bacilli were diminished during the treatment, nor was the virulence of the bacilli diminished.

On the use of creosote, Dr. F. H. Warner states that (*New York Medical Journal*, November 11th, 1893), during a somewhat extensive employment of this remedy in phthisis for the past four years, both in hospital and private practice, he has watched with great encouragement the steady gain in the results obtained; but it has been only during the latter half of the time that the positive value of creosote as an agent for combating most powerfully the effects of this disease has been apparent.

Formerly his custom was to administer the drug in small doses, exceptionally giving more than six or eight minims daily. During the last couple of years, however, the doses have been largely increased, with correspondingly better results. It is his rule to administer creosote in the following manner: Beginning with one minim three times daily, he increases one minim each day until the limit of tolerance is established, watching the effect of the drug upon the stomach and bowels, and causing a daily examination of the urine to be made.

The cases under observation in which creosote has been employed have, as the result of the treatment, presented certain features common to all and quite characteristic.

The general condition of the patient, as a rule, rapidly improves; in some cases the appetite is better, the cough at first becoming less during the daytime, while remaining.

In reference to the mode of administration of the drug, it has not seemed to make much difference. He generally orders it to be taken after meals in milk or sweetened water or wine, or in combinations with a bitter, such as cinchona or gentian, or in a little whiskey and water.

From the results obtained he has a strong belief in the present and future value of creosote in the treatment of pulmonary phthisis.

Griffith[5] states that (1) guaiacol, where used locally or internally, is a powerful antiseptic in tuberculosis; (2) tuberculous patients, to whom guaiacol is given internally, show marked increase in weight, strength, and appetite, if the use of the drug is continued long enough; (3) the exhibition of guaiacol in joint or bone tuberculosis should be continued through a long period of time; (4) guaiacol, unlike its close relation, creosote, is non-irritating to the stomach, and is well borne for a long period; and (5) guaiacol is a great aid to the surgeon in the treatment of all forms of tuberculosis of joints or bone.

Guaiacol has been given hypodermically by Peter[6], who refers also to the results obtained by Labadie-Lagrave, Picot, and Weill, all of whom have given the drug hypodermically with good results. Of twenty-five cases, Peter obtained benefit in all but three, which terminated in death. For hypodermic injection Picot's formula is as follows: Sterilised oil, 100 grammes; guaiacol, 5 grammes; iodoform, 1 gramme; and of this solution two to three cubic centigrammes are given daily.

In a recent issue of the *Medical Week* Professor R. Lépine presents the following conclusions regarding the endermic application of pure guaiacol: 1. External applications of guaiacol are likely to prove extremely useful in certain cases of pyrexia of tubercular origin. 2. The application of from one to two cubic centimetres is practically devoid of all risk of collapse, provided the tubercular process has not reached the state of suppuration and cavity-formation. In the latter case two grammes may cause death, as in a patient under Dr. Bard's observation. 3. Except in cases of extreme irritability of the skin, the application of pure guaiacol is never followed by any inflammatory reaction. 4. The only disadvantages of this method in the earlier stages of pulmonary tuberculosis are profuse sweating, with or without shivering, and a few other symptoms of a very mild description, so that this remedy is to be preferred to antipyrine, and even to acetanilide in many cases of phthisis, especially as it has no deleterious influence on digestion. Dr. Lépine agrees with Bard, that guaiacol is absolutely without effect on patients suffering from hectic fever due to successive exacerbations of the pneumonic process or to the presence of suppurating ulceration. On the other hand, it produces a powerful and lasting effect in cases of simple tubercular pyrexia, due to the formation of successive crops of granuloma; in short, wherever the fever is not due to septic infection superadded to the tubercular process. Under the influence of the drug cough and expectoration diminish, the patient sleeps better, the fever and night sweats cease, appetite returns, and the patient gains weight and strength. But the effect of the treatment must be watched, as the injections have been known to cause hæmoptysis, pneumonia, acute phthisis, and fat embolism. Untoward effects are to be feared when the taste of the drug remains long in the mouth, when the urine is black, or when the temperature rises to 40 deg. to 41 deg. centigrade, or falls to 35 deg. to 34 deg.

Turia[7] reports the results obtained in 18 cases of pulmonary tuberculosis by means of hypodermic injections of guaiacol and iodoform, in combination and separately, in Professor Bozzolo's clinic at Turin. The technique of the injections was as follows: The part, having been well washed with a 5 per cent. solution of carbolic acid, was then covered with a thin layer of wool saturated with the same solution, and on this was placed a small piece of ice, which was allowed to remain there for three or four minutes. This produced complete local anæsthesia. The injections were given, as a rule, in the post-trochanteric sulcus, or into the substance of the gluteal muscles, and in the whole series of 350 injections neither abscess nor other sign of local reaction was observed. The guaiacol used was of the purest quality, giving the characteristic purple reaction with sulphuric acid. Oil of sweet almonds was used as the excipient, as being less irritating and less liable to rancidity than vaseline. The author concludes from his observations that the method of treatment described is productive of no benefit in advanced cases, and is of little or no advantage even in the incipient stage.

In connection with this important question, Venturi[8] has approached the subject from a different point of view, and has tried to combat by means of drugs the action of the sterilised chemical products of the tubercle bacillus. He found that there was no constant appreciable difference between the duration of life and lesions of animals inoculated with the sterile cultures together with the drug, and those of control animals inoculated with the toxin only. Iodoform and guaiacol therefore exercised no retarding influence, whether injected separately or along with the toxin, nor even if the toxin had been mixed with either of them for a considerable time before being injected. With regard to the use of the drugs by the stomach, the author considers that they do more harm than good by deranging the digestive processes.

*Aristol* has been given subcutaneously in six cases of phthisis by J. Ochs[9] in the form of a 1 per cent. solution in oil of almonds. The injections were not toxic but were very painful, pain lasting throughout the day. The results were not very satisfactory.

*Salol.*—Grossi[10] has employed subcutaneous injections of salol with advantage. If we except one case which was already very advanced, in ten out of eleven cases the night sweats disappeared, the cough became less frequent, and the bacilli less abundant. He uses the salol in the following solution: Sweet oil of almonds, 30 grammes; salol, 10 grammes.

He gives five centigrammes in one injection. At the outset he gives one injection daily, and later on repeats the injection twice or three times daily, so as to give a total daily dose of five grammes of salol, *i.e.*, twenty grammes of solution. Injected under the skin the salol undergoes the well-known decomposition splitting into phenic and salicylic acids, and in twenty or thirty minutes the latter can be found in the urine. Grasset[11] has also given salol subcutaneously.

### Cough.

*Cough.*—The treatment of the cough of phthisical patients should depend on whether the cough is dry or accompanied by abundant expectoration. M. Maragliano[12] often treats those cases in which the expectoration is slight by preparations of opium. A very effective means of quieting the cough consists in the inhalation of chloroform in doses of ten to twenty drops. If the bronchial secretion is scanty, and at the same time very viscid, he recommends an atomiser with the following solution: Sodii bicarb, 1 to 2 grammes; hydrochlorate of morphine, ·05 grammes; aq., dest. 100 grammes, for four sprayings.

The dry cough increases in intensity, and becomes especially painful towards evening, and generally also during the febrile exacerbations. In these conditions one can do much to lessen the cough by the administration of antipyretic remedies; of these M. Maragliano gives phenacetein the first place.

The use of preparations of opium is contra-indicated when the expectoration is abundant. In these cases it is necessary to have resort to balsamics—guaiacol or creosote—given internally and in the form of inhalations. One may give the following with advantage: R. oxalate of cerium, ·05 to ·10 grammes; excip. Q. S. fiant pil. xl. Four to six pills to be taken daily. 1, *Les Nouv. Rém.* Jan. 24th, 1893.

He employs salol with oil thus: Sweet oil ℥i.; salol, ʒiij. He has obtained some satisfactory results.

Thorner[13] has found considerable benefit from the use of tuberculin. In advanced pulmonary tuberculosis he obtained improvement in the cough, night sweats, and in one case of laryngeal tuberculosis the improvement was very great.

1 Prac., Oct., 1893. 2 Lumleian Lectures, Brit. Med. Journ., 1893. 3 Ibid., Oct. 21, 1893. 4 Berl. Klin. Woch., No. 51., p. 1300. 5 Amer. Surg. and Gynœc., vol. iii., No. 9. 6 La France, Jan. 6, 1893; Gas. des Hop., No. 11, 1893. 7 Gas. deg. Osp., Oct. 1, 1892; Brit. Med. Journ., Jan. 28, 1893. 8 Lo Sperim., May 15, Brit. Med. Journ., Aug. 26, 1893. 9 Prag. Med. Woch., No. 36, 1822. 10 Med. Nev., No. 11, Les. Nouv. rém., May 24. 11 Journ de Méd. de Paris., June 11, 1893. 12 Les. Nouv. rém., Jan. 24, 1893. 13 Dent. Med. Woch., Sep. 14, 1893.

